# A Linguistically Informed Autosomal STR Survey of Human Populations Residing in the Greater Himalayan Region

**DOI:** 10.1371/journal.pone.0091534

**Published:** 2014-03-10

**Authors:** Thirsa Kraaijenbrink, Kristiaan J. van der Gaag, Sofia B. Zuniga, Yali Xue, Denise R. Carvalho-Silva, Chris Tyler-Smith, Mark A. Jobling, Emma J. Parkin, Bing Su, Hong Shi, Chun-Jie Xiao, Wen-Ru Tang, V. K. Kashyap, R. Trivedi, T. Sitalaximi, Jheelam Banerjee, Karma Tshering of Gaselô, Nirmal M. Tuladhar, Jean-Robert M. L. Opgenort, George L. van Driem, Guido Barbujani, Peter de Knijff

**Affiliations:** 1 MGC Department of Human and Clinical Genetics, Leiden University Medical Centre, Leiden, the Netherlands; 2 The Wellcome Trust Sanger Institute, Wellcome Trust Genome Campus, Hinxton, United Kingdom; 3 Department of Genetics, University of Leicester, Leicester, United Kingdom; 4 State Key Laboratory of Genetic Resources and Evolution, Kunming Institute of Zoology and Kunming Primate Research Centre, Chinese Academy of Sciences, Kunming, Yunnan, PR China; 5 Human Genetics Centre, Yunnan University, Kunming, Yunnan, PR China; 6 National DNA Analysis Center, Central Forensic Science Laboratory, Kolkata, India; 7 Himalayan Languages Project, Institut für Sprachwissenschaft, University of Bern, Bern, Switzerland; 8 Centre for Nepal and Asian Studies, Tribhuvan University, Kirtipur, Nepal; 9 Department of Life Sciences and Biotechnology, University of Ferrara, Ferrara, Italy; Erasmus University Medical Center, Netherlands

## Abstract

The greater Himalayan region demarcates two of the most prominent linguistic phyla in Asia: Tibeto-Burman and Indo-European. Previous genetic surveys, mainly using Y-chromosome polymorphisms and/or mitochondrial DNA polymorphisms suggested a substantially reduced geneflow between populations belonging to these two phyla. These studies, however, have mainly focussed on populations residing far to the north and/or south of this mountain range, and have not been able to study geneflow patterns within the greater Himalayan region itself. We now report a detailed, linguistically informed, genetic survey of Tibeto-Burman and Indo-European speakers from the Himalayan countries Nepal and Bhutan based on autosomal microsatellite markers and compare these populations with surrounding regions. The genetic differentiation between populations within the Himalayas seems to be much higher than between populations in the neighbouring countries. We also observe a remarkable genetic differentiation between the Tibeto-Burman speaking populations on the one hand and Indo-European speaking populations on the other, suggesting that language and geography have played an equally large role in defining the genetic composition of present-day populations within the Himalayas.

## Introduction

Until relatively recently, the reconstruction of human population prehistory was predominantly based on archaeology, linguistics, ethnography and somatology. In recent decades, DNA research has provided a new and powerful tool for studying mankind's prehistory. A geographical area of particular interest for human population prehistory is the Himalayas, stretching from Pakistan to Myanmar and forming the highest land boundary on our planet. There are several competing theories about the origin of the people currently residing in Asia in general and the Himalayan region in particular [1]-[5]. The geographical area comprising the present-day states of Nepal and Bhutan may have provided corridors for human migration in ancient times, resulting in relatively early inhabitation of the area. Alternatively, the region could have been rather inhospitable, and human survival could have been difficult, resulting in this area being one of the last parts of Asia to become populated following routes that are currently largely unknown.

Linguistically, the Greater Himalayan region is one of the most complex areas of the world. It contains populations speaking languages belonging to six different linguistic phyla (Austroasiatic, Altaic, Daic, Dravidian, Indo-European and Tibeto-Burman) and two confirmed linguistic isolates (Burushaski and Kusunda). This complex linguistic patchwork may be an indication of the Himalayas being an ancient source of genetically differentiated populations and languages which evolved *in situ*, a possible consequence of subdivision and extreme isolation over long periods of time. In addition to being one of the linguistically most complex regions, the Himalayas were sometimes thought to form the boundary between the Indo-European and Tibeto-Burman language phyla. In fact the real linguistic boundary, at least in present times, runs roughly parallel to and well south of the highest mountain peaks, through the foothills and lowlands [1].

Regardless which settlement theory will prove to be correct, there can be no doubt that the amount of (pre)historic geneflow between people residing in and around the Greater Himalayan Region must have been influenced substantially both by the large linguistic diversity and by the rugged terrain which, even with modern means, still renders large parts of the region very difficult to traverse. Previous genetic studies, mostly focussing on the Y-chromosome or the mtDNA, have indeed indicated the possible presence of a genetic boundary in this area. But so far, these studies have mainly included population samples from countries surrounding the Himalayas or at most, a small, poorly defined, Nepalese population sample [4], [6]–[10] and have, therefore, been unable to pinpoint the most likely location of this boundary, if it exits. A detailed and linguistically-informed genetic study of populations residing in the Himalayan heartland is needed in order to determine whether geography or language has, historically, had a more substantial influence on geneflow. Nepal and Bhutan hug the ethnolinguistically and topographically complex Southern flank of the mountain range, covering the area of the boundary between the Indo-European and Tibeto-Burman phyla and the mountain passes most likely to have served as migratory corridors. The current study compares genotypic data for 15 highly polymorphic autosomal STRs from populations living in Nepal and Bhutan with those of the countries surrounding the Greater Himalayan Region. Relatively small numbers of autosomal STRs have proven effective in the past for distinguishing between populations of different linguistic affiliations [11]–[13], although not in a completely comparable geographical setting. Therefore, we hope the use of autosomal STRs in the current study will at least provide valuable insights into the influence of both linguistics and geography on the (pre)historic genetic differentiation of Asian populations in general and those of the Greater Himalayan Region in particular and may even provide some indications regarding the settlement issue.

## Materials and Methods

### Sample collection and consent

For the purpose of the EUROCORES collaborative project 'Language and Genes of the Greater Himalayan Region' we have collected blood samples from Nepalese and Bhutanese volunteers over the age of 18. These DNAs are anonymous and identified only by code. Collection of these blood samples took place in Nepal and Bhutan. All subsequent research using these samples was done in the UK, Italy, and the Netherlands. Prior to sample collection, the entire project was submitted to the LUMC Medical Ethics Committee. Since this project was strictly non-commercial, non-medical and a non-intervention study, samples would be obtained under full consent, and identified by code only, and of adult (>18) individuals, the LUMC MEC did not further process this project, which was in line with the relevant version of the Dutch Guidelines of Medical Research. Hence no approval was necessary, and not obtained.

A detailed description of the entire project, including sample collection, study design and consent structure and acknowledgements is available via www.le.ac.uk/ge/maj4/Himalayan_OMLLreport.pdf.

In short, sampling took place according to the general guidelines of the human global diversity project, and full written informed consent was obtained from each individual as was agreed by bilateral agreements between Leiden University Medical Center and the responsible authorities in Nepal and Bhutan. Individuals were approached via representatives of the respective language communities and the purpose of the project was explained in local languages. Consent forms to be signed included a condensed text of the sampling guidelines. When a donor was unable to read or write, the consent text was read to the donor in his/her local language, after which one of the project's co-workers filled in the donor's data. For some communities, detailed explanation in the local language was given and video-recorded for archival purposes. All consent forms are archived at Leiden University Medical Center.

Our aim was to collect samples from approximately 50 unrelated representatives of each of the major ethnolinguistic groups present in Nepal and Bhutan [1], [14].

Between December 2002 and February 2003, blood samples were collected from 947 unrelated Nepalese volunteers (764 males and 183 females, table 1), belonging to 41 ethnolinguistic groups from the Tibeto-Burman phylum and 15 ethnolinguistic groups from the Indo-European phylum. To ascertain that a person belonged to a certain ethnolinguistic group or caste, only volunteers with both parents and all four grandparents belonging to the same group were sampled. Furthermore, before sampling, the donor's name and place of birth were systematically checked against what is known about the names adopted by members of Nepal's diverse ethnolinguistic groups and the geographical spread of these groups. In addition, several team-members spoke one or more relevant Nepalese languages, providing the opportunity to evaluate the donor's linguistic background.

**Table 1:** Group names and numbers of samples collected in Nepal and Bhutan.

Group/Pool^1^	Country	Linguistic phylum and -cluster^2^	n males	n females	Coordinates used in spatial analyses	Code^3^
**Artisanal caste Indo-Aryan**	**Nepal**	**IE, Eastern Pahādī**	**26**	**14**	**28,75 N/80,5 E**	**ACI**
Bahun (Brahmin)	Nepal	IE, Eastern Pahādī	25	8	29,1667 N/81,1667 E	BHU
Barām	Nepal	TB, Newaric	32	6	28,0667 N/84,6667 E	BAR
Black Mountain Mönpa	Bhutan	TB, East Bodish	40	18	27,2167 N/90,2167 E	MON
Bodo	N-India	TB, Brahmaputran	37	2	26,6667 N/90,3333 E	BOD
Brokkat	Bhutan	TB, Central/South Bodish	24	5	27,7333 N/90,4333 E	KAT
Brokpa (Bj'op)	Bhutan	TB, Central/South Bodish	40	10	27,4 N/91,7167 E	BRP
Bumthang	Bhutan	TB, East Bodish	50	10	27,6667 N/90,55 E	BUM
**Central Kiranti**	**Nepal**	**TB, Kiranti**	**42**	**6**	**27,1333 N/87,0458 E**	**CKI**
Chali	Bhutan	TB, East Bodish	50	11	27,3833 N/91,0167 E	CHL
Chantyal	Nepal	TB, Tamangic	21	2	28,4 N/83,3667 E	CHN
Chepang (Praja)	Nepal	TB, Magaric	20	7	27,5833 N/84,7 E	CHP
Chetri (Kshetriya)	Nepal	IE, Eastern Pahādī	37	10	29,1667 N/81,2 E	CHE
Dakpa (Dwagspo)	Bhutan	TB, East Bodish	49	10	27,4667 N/91,5167 E	DAK
**Danuwar & Kachadiya Danuwar**	**Nepal**	**IE, Eastern Pahādī, IA with suspected TB substrate**	**33**	**6**	**27,25 N/85,75 E**	**DKD**
Dhimal	Nepal	TB, Dhimalish	20	2	26,5 N/87,7 E	DHI
Dura	Nepal	TB	27	8	28,2833 N/84,2 E	DUR
Dzala	Bhutan	TB, East Bodish	51	11	27,9 N/91,15 E	DZA
**Eastern Kiranti**	**Nepal**	**TB, Kiranti**	**12**	**7**	**27,1389 N/87,4278 E**	**EKI**
Ghale	Nepal	TB, Tamangic	17	8	28,2833 N/84,7333 E	GHL
Gongduk	Bhutan	TB	46	10	27,0833 N/90,9333 E	GNG
Gurung	Nepal	TB, Tamangic	40	6	28,3 N/84,1167 E	GUR
High caste Newar	Nepal	TB, Newaric	24	6	27,6167 N/85,4333 E	HCN
Kham (Magar)	Nepal	TB, Magaric	13	1	28,5 N/83 E	KHM
Khengpa	Bhutan	TB, East Bodish	52	10	27,1333 N/90,6833 E	KHG
Kumal	Nepal	IE, Eastern Pahādī, IA with suspected TB substrate	21	5	28,05 N/84,45 E	KUM
Kurtöp	Bhutan	TB, East Bodish	51	13	27,8167 N/90,8167 E	KUR
Lakha	Bhutan	TB, Central/South Bodish	50	10	27,6833 N/90,15 E	LAK
Layap	Bhutan	TB, Central/South Bodish	25	5	28,0667 N/89,6833 E	LAY
Lhokpu (Lhop, Doya)	Bhutan	TB	39	8	26,95 N/89,1167 E	LHP
**Limbu**	**Nepal**	**TB, Kiranti**	**56**	**7**	**27,19 N/87,8333 E**	**LIM**
Magar	Nepal	TB, Magaric	40	6	28,0833 N/83,8333 E	MGR
Majhi (Bote)	Nepal	IE, Eastern Pahādī, IA with suspected TB substrate	21	6	27,8333 N/83,6667 E	MAJ
Mangde ('Nyenkha, Henke)	Bhutan	TB, East Bodish	54	10	27,4167 N/90,2167 E	MNG
Newar	Nepal	TB, Newaric	44	10	27,6167 N/85,4 E	NWR
'Ngalop (Dzongkha)	Bhutan	TB, Central/South Bodish	50	10	27,5333 N/89,4833 E	NGA
Nup (Trongsap)	Bhutan	TB, East Bodish	27	10	27,5833 N/90,3333 E	NUP
Sherpa (Solu-Khumbu)	Nepal	TB, Central/South Bodish	20	5	27,7333 N/86,5833 E	SHE
Tamang	Nepal	TB, Tamangic	41	9	27,8833 N/85,4167 E	TMG
Thakali	Nepal	TB, Tamangic	20	9	28,8167 N/83,75 E	THK
Thangmi	Nepal	TB, Newaric	16	2	27,75 N/86 E	THG
Tharu	Nepal	IE, Maithili & Bhojpuri, IA with suspected TB substrate	28	7	27,4167 N/83,3333 E	THR
Toto	N-India	TB, Dhimalish	54	16	26,6667 N/89 E	TOT
Tshangla (Shâchop)	Bhutan	TB	50	11	27,1833 N/91,3167 E	TSH
**Western Kiranti**	**Nepal**	**TB, Kiranti**	**51**	**14**	**27,3833 N/86,6 E**	**WKI**

1 Names of pools are shown in bold print, and names of groups in normal print. Alternative group-names are shown between brackets. For information about the populations included in the pools, see table S2.

2 Classification according to van Driem [1]; TB: Tibeto-Burman, IE: Indo-European, IA: Indo-Aryan. For more detailed cluster data, see figure S1.

3 Codes used in figures to indicate the populations.

During five expeditions between October 2003 and February 2004, blood samples were collected from 920 unrelated Bhutanese volunteers, belonging to 17 ethnolinguistic groups of the Tibeto-Burman phylum (839 males and 187 females, table 1). Prior to the expeditions, the ethnicity of all volunteers was identified by local representatives of the Dzongkha Development Authority (DDA) of Bhutan. During the expeditions in Bhutan, we were also able to collect blood samples from 109 unrelated volunteers (91 males and 18 females, table 1) belonging to two Tibeto-Burman speaking populations from Northern India: the Bodo and the Toto, who regularly cross the Indian-Bhutanese border.

**Figure 1:**
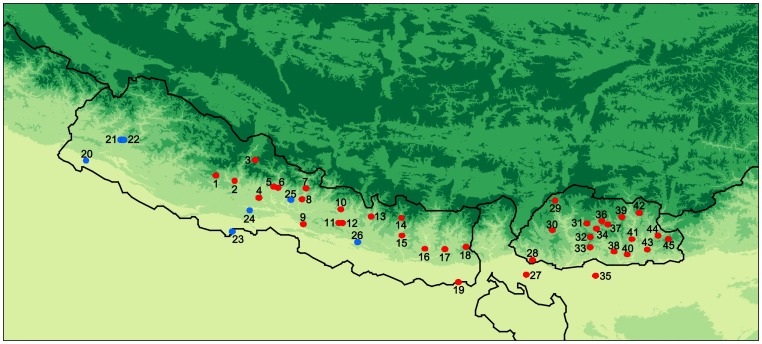
Distribution of ethnolinguistic groups/pools sampled in Nepal and Bhutan. Blue dots indicate the approximate geographical centres of the Indo-European groups/pools and red dots indicate the approximate geographical centres of the Tibeto-Burman groups/pools. Populations from Nepal: 1 = Kham, 2 = Chantyal, 3 = Thakali, 4 = Magar, 5 = Dura, 6 = Gurung, 7 = Ghale, 8 = Barām, 9 = Chepang, 10 = Tamang, 11 = Newar, 12 = High caste Newar, 13 = Thangmi, 14 = Sherpa, 15 = Western Kiranti (pool), 16 = Central Kiranti (pool), 17 = Eastern Kiranti (pool), 18 = Limbu (pool), 19 = Dhimal, 20 = Artisanal caste Indo-Aryan (pool), 21 = Bahun, 22 = Chetri, 23 = Tharu, 24 = Majhi, 25 = Kumal, 26 = Danuwar & Kachadiya Danuwar (pool). Populations from Bhutan and India: 27 = Toto (India), 28 = Lhokpu, 29 = Layap, 30 = 'Ngalop, 31 = Lakha, 32 = Mangde, 33 = Black Mountain Mönpa, 34 = Nup, 35 = Bodo (India), 36 = Brokkat, 37 = Bumthang, 38 = Khengpa, 39 = Kurtöp, 40 = Gongduk, 41 = Chali, 42 = Dzala, 43 = Tshangla, 44 = Dakpa, 45 = Brokpa. © John Benjamins Publishing Company, Amsterdam/Philadelphia.

Figure 1 shows the geographical spread of the Himalayan populations examined in this study: for each population the approximate geographical centre of their original area of residence is depicted (the coordinates of these centres are given in table 1). Figure 1 has previously been published in ref. [15] and has been re-used in the current publication with kind permission by John Benjamins Publishing Company, Amsterdam/Philadelphia (http://www.benjamins.com, last accessed 2014 Feb 14).

### Reference samples from surrounding countries

In order to be able to perform detailed comparative statistics, reference data from countries surrounding the Greater Himalayan Region were necessary. Our aim was to collect as many representatives as possible of all linguistic phyla present in this region.

DNA samples from two Dravidian-speaking Indian populations [16] and from the Asian populations present in the HGDP-CEPH panel (belonging to several linguistic phyla) [17], [18] were available for autosomal genotyping at the Forensic Laboratory for DNA research.. In addition, autosomal genotypes from 76 Indian and Chinese populations [19]–[26], [31] and DNA-samples from another 40 populations residing in several countries north and south of the Himalayas [9], [16], [27]–[30] were made available from the co-authors’ sample collections. The linguistic isolate Burusho (HGDP-CEPH panel) was not included in our analyses.

In this way, we collected genetic reference data from a total of 6,899 people, representing 6 Austroasiatic populations, 23 Altaic populations, 4 Daic populations, 33 Dravidian populations, 5 Hmong-Mien populations, 37 Indo-European populations and 34 Tibeto-Burman populations, including 6 Tibeto-Burman populations from India (supporting information (SI) table S1). For many reference populations, names were given in the original researchers' native language instead of English. In this paper, we have chosen to use English names for all populations. When alternative spellings are regularly used for a certain population or a population was originally indicated by a non-English name, these alternative names are shown between brackets in table S1. When the original researchers did not specify to which linguistic phylum a reference population belonged, the most likely linguistic phylum was determined based on the specifications of van Driem [1] and/or the Ethnologue [14].

### DNA isolation and genotyping

DNA from the Himalayan blood samples was isolated using Autopure LS® from Gentra Systems, according to the manufacturer's specifications.

After DNA isolation, all Himalayan samples were genotyped for 21 forensic autosomal STRs, contained in three commercially available multiplex PCR kits: Powerplex®16 (Promega), AMPF*l*STR® Identifiler® (Applied Biosystems) and FFFL® (Promega). PCR amplification was performed according to the manufacturers' specifications, but using a total reaction volume per sample of 12.5 µl instead of 25 µl. PCR products were analysed using an ABI3100 automated DNA sequencer and the Genemapper®ID software.

The autosomal genotypes of 66 [9], [16]–[18], [27]–[30] out of the 142 reference populations collected, have been determined in the current study. Since the autosomal genotypes from the collection of V.K. Kashyap were mostly limited to the 15 highly informative STRs contained within the Powerplex®16 kit and further genotyping of these samples was currently not feasible, we limited the genotyping of the other reference populations to the Powerplex®16 kit as well, with the exception of the reference populations from Yunnan, China which were genotyped for the 21 forensic autosomal STRs previously [31].

The overall allele frequency data for Nepal and Bhutan have been published previously [32], [33], as have the allele frequency data for many of the Indian reference populations [19]–[26]. Genotypes and allele frequency data for the reference populations from Yunnan, China are available online [31]. For allele frequency data from the other reference populations [9], [27]–[30] or for previously unpublished genotype data, please refer to the original researchers.

### Statistical analyses

Allele frequencies and summary statistics were calculated using a combination of the Excel add-ins Microsatellite Toolkit [34] and GenAlEx 6.5 [35], [36]. Average within-group relatedness values were determined using the software STORM 2.0 [37], which applies a calculation method based on the method described by Li et al. [38]. In addition, the value of 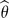, as described by Friedlaender et al. [39], was calculated for all populations. In order to test for significant differences, 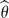 values were submitted to a non-parametric Kruskal-Wallis test, followed by Dunn's multiple comparison test. The results of these tests were visualised using GraphPad Prism 6 [40].

Since only a small number of reference populations could be collected for the phyla Austroasiatic, Daic and Hmong-Mien (6, 4 and 5 respectively), we excluded these phyla from all statistical analyses in order to prevent sampling bias influencing the results. Furthermore, we decided to treat the 6 Tibeto-Burman speaking Indian populations as Himalayan test populations rather than reference populations, in order to investigate the possibility of (historical) admixture with Indo-European and/or Dravidian speakers from India.

The presence or absence of population (sub)structure within the Himalayas was examined using the following analysis methods:

1. In order to test for the possible presence of a genetic boundary, a pairwise *F*_ST_ matrix for all population pairs was generated using GenAlEx 6.5 and analysed in the program Barrier vs 2.2 [41].

2. Admixture estimations for the Himalayan populations were generated using a weighted least squares approach in the program Admix95 [42]. The parental populations were chosen according to linguistic affiliation; Tibeto-Burman, Indo-European, Dravidian and Altaic and for the test-populations the Himalayan populations were grouped into Nepalese Tibeto-Burman speakers, Nepalese Indo-European speakers, Bhutanese Tibeto-Burman speakers and Indian Tibeto-Burman speakers.

3. The dataset was further examined in the program STRUCTURE 2.3.2 [43]–[46] using the admixture model with correlation between allele frequencies across clusters, both with and without use of the sampling locations as priors. The number of clusters (K) investigated ranged from 2 to 6, and for each K, ten independent STRUCTURE runs were performed, all using a burn-in of 20,000 iterations, followed by 10,000 iterations of MCMC for estimates of clustering.

4. In order to verify the STRUCTURE results, the dataset was subsequently examined using the Excel-based program FLOCK 3.0 [47], [48], which makes use of a non-Bayesian method for the estimation of admixture coefficients. In accordance with the STRUCTURE analyses, FLOCK analyses were also performed for 2 to 6 clusters, using the default parameter settings as provided by the program.

5. The *F*_ST_ matrix as generated by GenAlEx 6.5 was used both in a principal coordinates analysis (PCoA) within GenAlEx 6.5 and in a multi-dimensional scaling analysis (MDS) using the program NCSS97 [49]. The first two dimensions resulting from the NCSS analysis were used for creating an MDS plot in Excel.

6. Using the R script CHROMA, kindly provided by Jeff Long [50], we made a comparison of the within- and between-population variation based on gene identity values (i.e. the chance that two randomly chosen copies of a locus, either from the same population or from two different populations, are identical by state). In general, it can be assumed that pairs of populations showing a relatively high gene identity will be more closely related than populations showing low gene identity.

7. Subsequently, the genotype data were analysed in Bottleneck 1.2.02 [51] in order to test for the possible occurrence of recent bottlenecks.

8. Pairwise matrices for linguistic, geographical and genetic distance, both on the population level (165*165) and on the individual level (7415*7415), were analysed using the software tool zt [52] to perform two types of Mantel tests:

  - A simple Mantel test, which determines the classical Pearson correlation coefficient (r) and corresponding p-value for pairs of distance matrices.
  - A partial Mantel test, which determines the correlation between two distance matrices while controlling the effect of a third matrix, in order to remove spurious correlations.

In both types of Mantel tests, the null hypothesis is that the distances in the different matrices are independent. On the population level, both the simple and partial Mantel tests were performed with 10,000, 100,000 and 1 million randomizations, however, this did not affect the resulting correlation coefficients. On the individual level, only 10,000 randomizations could be tested, due to limitations of computational capacity. In order to get an indication of the correlations both within and across linguistic phyla, all Mantel tests were performed on the dataset as a whole and on 4 subsets; one for each phylum.

The distance matrices used in the Mantel tests were constructed as follows:

For genetic distance between populations, we constructed an *F*_ST_/(1- *F*_ST_) matrix [53], using the *F*_ST_ matrix previously generated in GenAlEx 6.5. For individuals, a pairwise dissimilarity matrix was constructed. For this purpose we first determined pairwise similarity by counting the number of shared alleles and dividing this number by the total number of alleles compared. Subsequently, genetic dissimilarity was calculated as 1-similarity.

The matrices for geographical distance, measured 'as the crow flies', were constructed using the Geographic Distance Matrix Generator (GDMG) version 1.2.3 [54], with the coordinates given in table 1 and table S1. In the matrix at the individual level, individuals belonging to the same population were assigned a virtual distance of 0.05 km. We are aware that in a mountainous landscape such as the Himalayas, distance 'as the crow flies' (which does not take landscape features into account) is not the best representation of reality. We therefore would have preferred to use a matrix of distances expressed as 'days walking', but found this an impossible feat to manage for all populations included in the study. Since we were also unable to find a method to effectively assign some form of penalty to the distances when traversing the Himalayas, and since for the populations living outside the Himalayas, distance 'as the crow flies' can be assumed to be representative, we decided to use this distance for all populations.

For the linguistic distance matrices, the information as given by van Driem [1] and/or the Ethnologue [14] was used to connect the Himalayan populations and the reference populations into four linguistic networks (one for each phylum).

For the Tibeto-Burman, Altaic and Dravidian populations, the construction of these linguistic networks was straightforward, since these linguistic phyla have been extensively studied and the subclassifications within them are mostly undisputed. However, we would like to register the following proviso regarding these networks: The ethnolinguistic identification of our Himalayan populations has been rigorous and for most of the reference populations in this study, the ethnolinguistic identification of the populations as given by the original researchers is also unproblematic. However, for some of the reference populations, adequately specific ethnolinguistic information was not provided, leaving us with no other option than to assume the most likely linguistic background for these populations based on their region of origin.

Regarding the Indo-European network, in addition to the proviso given for Tibeto-Burman, Altaic and Dravidian, we here clarify some of our choices:

a. Indo-Aryan phylogeny is a relatively neglected field. Whilst methodologically-rigorous historical linguistic comparisons have been devoted to Eastern Indo-Aryan phylogeny, e.g. Chattterji [55], Majumdar [56], and especially Pattanayak [57], the historical phonological evidence for the affiliation of Gujarati has been interpreted by some scholars as supporting a 'southwestern' affiliation to Marathi and Konkani, whereas other regular phonological changes, e.g. the sound law s. > kh, clearly support a more northerly Śaurasenī affiliation, e.g. Hoernle [58], Pandit [59]–[61]. Turner argued that the language of the A<$>\raster(50%)="rg1"<$>okan inscription at Girnar, dating from the third century BC, resembled an earlier form of Marathi rather than early Gujarati. Therefore, the ancestral speakers of Gujarati once lived further north and must have pushed Proto-Marathi speakers southward (Turner [62], [63]). We find possible justification in Gujarati verbal morphology for grouping Gujarati with Nepali, Western Pahādī and Eastern Pahādī in an Eastern Śaurasenī group, cf. Hoernle [58], Turner [64] and Joshi [65]. This new phylogenetic proposal deviates somewhat from Rudolf Hoernle's original Indo-Aryan phylogeny, however. With Western Śaurasenī or 'Midland Indo-Aryan' representing a posterior eastward diffusion downstream along the Gangetic plain, the historical linguistic model implied here presumes several discrete waves of linguistic diffusion eastward across Northern India instead of Hoernle's inner vs. outer model consisting of just two waves of Indo-Aryan incursion.

b. Within the network, there are two nodes classified as Indo-European with suspected Tibeto-Burman substrate. This classification is based on the widely perceived phenotypical similarity of these populations with Tibeto-Burman speaking populations of Nepal. Furthermore, their languages contain features such as biactantial verbal agreement or otherwise peculiar morphology in the Indo-European context. A widely held but so far untested explanation for these traits has been to attribute a Tibeto-Burman origin to these populations who must have, in that case, adopted their current Indo-European language long ago.

Subsequently, these four networks were combined into one network of Asian languages (figure S1). Based on the findings reported by Greenhill et al. [66] and on findings by van Driem (personal communication), it can be assumed that distances between different linguistic phyla are substantially larger than distances within linguistic phyla. Therefore, branch lengths of >1 were assumed for the branches connecting the four phyla, keeping in mind that some phyla are likely to be more closely related than others (as indicated by the numbers next to the corresponding branches in figure S1). This final network was used to construct the linguistic distance matrices by counting the number of nodes separating each pair of populations (individuals belonging to the same population were assigned a virtual linguistic distance of 1 in the matrix on individual level).

9. In order to verify the zt results and to test for possible isolation by distance effects on the genetic composition of our dataset, the genetic, geographic and linguistic distance matrices constructed for analysis in zt were further analysed using the Isolation By Distance Web Service (IBDWS) version 3.23 [67] with the default analysis parameters as provided by the web service.

## Results and Discussion

### Sample collection and genotyping

In total, we collected DNA samples from 947 unrelated Nepalese volunteers, 920 unrelated Bhutanese volunteers and 109 unrelated North-Indian volunteers, belonging to a total of 60 ethnolinguistic groups from the Tibeto-Burman phylum and 15 ethnolinguistic groups from the Indo-European phylum (table 1). For many of the Nepalese groups, only small numbers of individuals could be sampled. Therefore, small groups belonging to the same linguistic cluster (as determined by van Driem [1]) were pooled for statistical analyses in order to minimize sample size bias (274 individuals in total; table S2A). Small groups which could not be pooled effectively (23 individuals in total; table S2B) were included in the genotyping, but were excluded from all statistical analyses, leaving 924 Nepalese individuals, subdivided into 19 Tibeto-Burman groups/pools and 7 Indo-European groups/pools (table 1) for statistical analyses.

**Figure 2:**
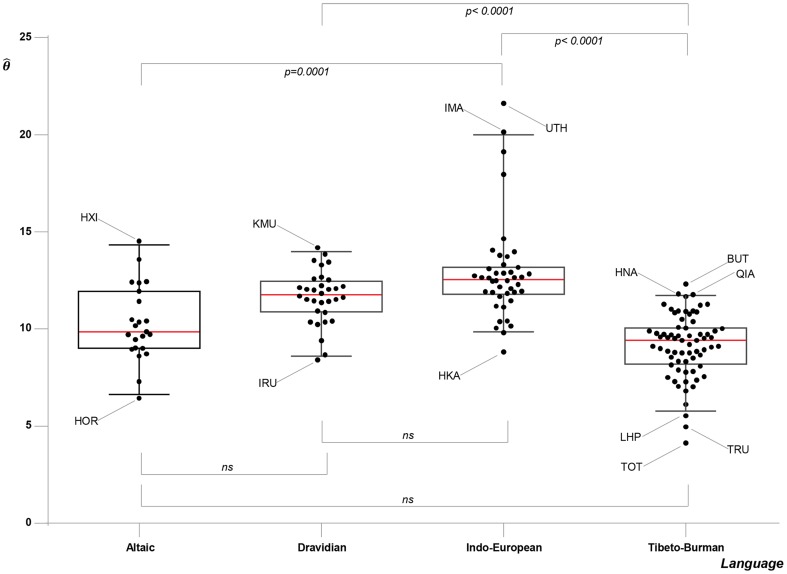
Results of significance tests for 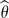 values. 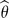
 values are grouped by linguistic phylum with each dot representing one population. The median per phylum is indicated by a red line, the boxes surrounding the medians indicate the 25^th^ and 75^th^ percentiles and the 5^th^ and 95^th^ percentiles are indicated as error-bars. Non-significant results of the Kruskal-Wallis/Dunn's multiple comparison test are indicated by lines with the text *ns* below the 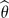 values and significant results are indicated by lines with p-values above the 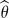 values. For the population abbreviations used in the figure, see tables 1 and S1.

General allele frequency data for the Nepalese and Bhutanese populations were published previously [32], [33]. Full genotype data and detailed allele frequency data for each ethnolinguistic group/pool are available upon request from the corresponding author. We analysed these new data together with 5795 individuals (120 populations) from surrounding regions, mostly derived from published sources (Materials and Methods). All statistical analyses described below are thus based on comparisons of the Himalayan populations with 35 Indo-European, 30 Tibeto-Burman, 32 Dravidian and 23 Altaic reference populations. Summary statistics, average relatedness values and 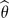 values for all 165 populations included in this study are given in table S3. On average, the 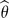 values obtained for Altaic and Tibeto-Burman populations are lower than the 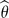 values obtained for Indo-European and Dravidian populations (figure 2), indicating that effective population sizes for Altaic and Tibeto-Burman populations may, in general, have been smaller than those of Indo-European and Dravidian populations [39]. Upon comparison through a non-parametric Kruskal Wallis test, followed by Dunn's multiple comparison test, the 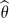 values of three pairs of linguistic phyla were found to show significant differences: Tibeto-Burman vs Indo-European (p<0.0001), Tibeto-Burman vs Dravidian (p<0.0001) and Altaic vs Indo-European (p = 0.0001). The other pairs did not show significant differences.

### Analyses of gene flow

In order to explain the current genetic situation in the Greater Himalayan Region, we assumed that one the following three theories must be true:

1. Geographical distance has played a more important role than linguistic differences in the occurrence or absence of (pre)historical population interactions.

2. Linguistic differences played a more important role than geographical distance in the occurrence or absence of (pre)historical population interactions.

3. Geography and linguistics played similar roles in the occurrence or absence of (pre)historical population interactions.

These theories lead to the following predictions for the genetics of the Himalayan populations:

  - If theory 1 is correct, the genetic composition of the Himalayan populations, all residing to the south of the geographical boundary, should show the closest resemblance to the genetic composition of the Southern reference populations, independent of the language they speak.
  - If theory 2 is correct, we would expect the genetic composition of the Indo-European Himalayan populations to resemble that of the Indo-European reference populations most closely, with a corresponding resemblance between the Tibeto-Burman Himalayan populations and the Tibeto-Burman reference populations. The latter despite the fact that they reside on opposite sides of the geographical boundary.
  - If theory 3 is correct, the genetic composition of the Himalayan populations can resemble that of any or none of the reference populations.

Since the aim of the current study was to try to pinpoint a possible genetic boundary assumed to be present in the Himalayan populations, we first analysed our data in Barrier vs2.2. However, other than drawing two circular barriers isolating the Lhokpu and the Dora from all other populations, no barriers were indicated by the program. From this we concluded that if genetic differences exist between the Himalayan populations of different linguistic affinities, which is still very likely in view of the results of previous studies, they are, at least with the current (limited) autosomal STR dataset, too subtle to be translated into a clear boundary.

Admixture proportions were determined for the populations Nepal Tibeto-Burman, Nepal Indo-European, Bhutan and India Tibeto-Burman in order to investigate the genetic contribution by each of the parental populations Indo-European, Tibeto-Burman, Dravidian and Altaic (table 2). For the Nepalese Tibeto-Burman speakers and for the Bhutanese populations, a clear major contribution from the Tibeto-Burman parental population was observed (62.44% and 57.5% respectively), whereas the contribution of the Tibeto-Burman parental population to the Nepalese Indo-European speakers was only 13.42%. These results suggest that large scale historical gene flow across the Himalayan Mountains has indeed occurred and may have shown a linguistically informed pattern. The major contributor in the Nepalese Indo-European speakers was the Dravidian parental population (42.61%). Most present-day Dravidian speakers reside far to the South of the Himalayas, making recent large scale gene flow events an less likely explanation for this large admixture percentage. A shared ancestry between Nepalese Indo-European speakers and Dravidian speakers seems to be a more likely explanation. Since Dravidian is generally believed to be the aboriginal linguistic phylum of Southern Asia, this observation may lend support to the theory that, at least the Nepalese lowlands (where most Nepalese Indo-European speakers reside) could have been among the areas of earliest settlement in the Himalayan region.

**Table 2:** Summary of Admixture analysis.

	Nepal TB	Nepal IE	Bhutan	India TB
**IE (parental)**	0.0482±0.0554	0.2047±0.0544	0.1565±0.0229	–0.4276±0.4911
**TB (parental)**	0.6244±0.0490	0.1342±0.0424	0.5750±0.0181	1.1053±0.3807
**DR (parental)**	0.1711±0.0374	0.4261±0.0562	0.0203±0.0209	0.8141±0.3815
**AL (parental)**	0.1563±0.0000	0.2350±0.0000	0.2482±0.0000	–0.4917±0.0000

IE: Indo-European, TB: Tibeto-Burman, DR: Dravidian, AL: Altaic.

For the Indian Tibeto-Burman speakers the Admixture analysis resulted in negative admixture values for the Indo-European and Altaic parental populations, contrasted by an admixture value of >100% for the Tibeto-Burman parental population, suggesting that the model of 4 parental populations is not applicable to this population. Unfortunately, several alternative scenarios with only two parental populations also resulted in negative values for one of the two parental populations (not shown). Therefore, no useful admixture estimates could be obtained for the Indian Tibeto-Burman speakers.

### Analyses of population (sub)structure

**Figure 3:**
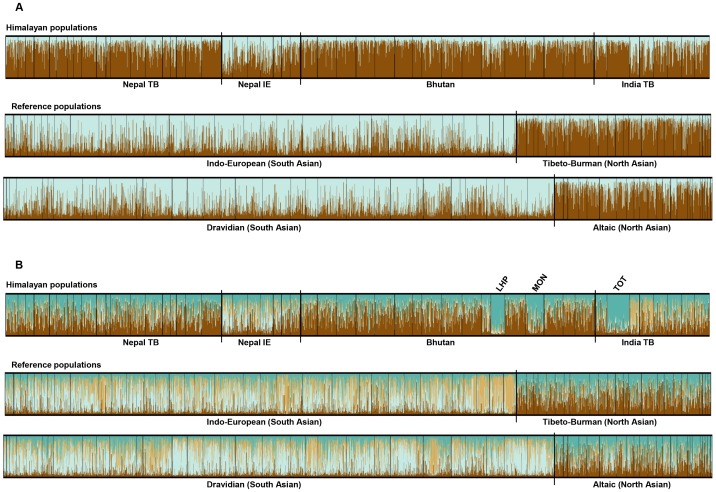
Results of STRUCTURE analyses. The colours represent the proportion of inferred ancestry from K ancestral populations. 3A: Unsupervised run for K = 2. The distribution pattern of inferred ancestry seems to indicate the presence of subtle population substructures within the Himalayas, with the Indo-European speaking Himalayan populations clustering more closely to the Indo-European and Dravidian reference populations (predominantly light blue) and the Tibeto-Burman speaking Himalayan populations clustering more closely to the Tibeto-Burman and Altaic reference populations (predominantly brown). 3B: Unsupervised run for K>2. The clustering pattern as observed for K = 2 in the Himalayan populations is mostly lost in favour of the separation of the Lhokpu (LHP), Black Mountain Mönpa (MON) and Toto (TOT), although some differences can still be observed between the Tibeto-Burman and Indo-European populations (this figure shows the results for K = 4).

**Figure 4:**
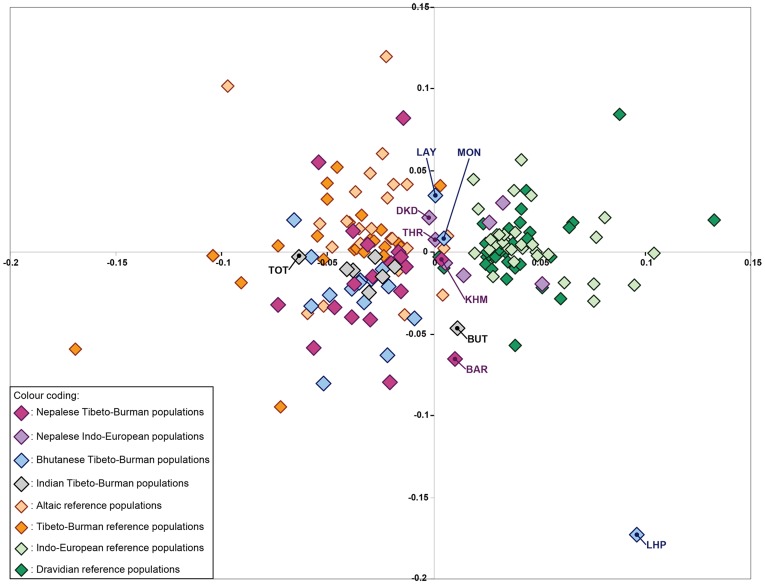
MDS plot. Populations have been colour coded according to linguistic phylum and geographical origin, as is explained within the figure. The population-codes used in this plot are explained in table 1 and table S1. The stress values for the dimensions used to construct this plot were 0.333475 and 0.216317 respectively.

In order to investigate the possibility of more subtle genetic differences, which might still give some indication toward the likelihood of the three theories stated above, we subsequently analysed the data with STRUCTURE 2.3.2 (the results of which are illustrated in figure 3), MDS (figure 4), FLOCK 3.0 (Figure S2), PCoA (figure S3) and CHROMA (Figure S4).

In these analyses, the first, easy to make, observation is that a distinction can be made between the Himalayan Indo-European speakers and the Himalayan Tibeto-Burman speakers:

  - When analysing the data in STRUCTURE 2.3.2, all runs for K = 2 showed a distribution pattern as in figure 3A. The populations belonging to the Indo-European and Dravidian phyla cluster together (predominantly light blue in figure 3A) while the populations belonging to the Tibeto-Burman and Altaic phyla cluster together in a second cluster (predominantly brown in figure 3A). A very similar clustering pattern is observed when the data are analysed for K = 2 by means of FLOCK 3.0 (figure S2A).
  - In the MDS analysis (figure 4), all but two of the Himalayan Indo-European populations co-localize with the Southern reference populations (Indo-European and Dravidian) on the right side of the Y axis, whereas all but six of the Himalayan Tibeto-Burman populations co-localize with the Northern reference populations (Tibeto-Burman and Altaic) on the left side of the Y axis. The PCoA shows a similar pattern for the distribution of the Himalayan Tibeto-Burman populations (figure S3). The pattern for the Himalayan Indo-European populations in the PCoA is slightly less distinct than that in the MDS: they are still clearly distinguished from the Himalayan Tibeto-Burman populations, but they don't co-localize as closely with the Southern reference populations.
  - In the CHROMA analysis (figure S4), the colour coding pattern for the gene identity values shows a subdivision into three areas:

1. A mostly green area (indicating relatively high gene identity values), containing the gene identity values for all pairwise comparisons between the Altaic and Tibeto-Burman reference populations and the Tibeto-Burman speakers from Nepal and Bhutan.

2. A transition zone with approximately equal amounts of blue and green, containing the values for the comparisons of the Tibeto-Burman speakers from India and the Indo-European speakers from Nepal to each other and the populations mentioned under 1.

3. A mostly blue area (indicating lower gene identity values), containing the values for the comparisons of the Indo-European and Dravidian reference populations to all other populations.

Since in all these observations the Himalayan Tibeto-Burman speakers show higher degrees of similarity to the Northern reference populations than to the Southern reference populations, the results seem to support a linguistic rather than a geographical subdivision for the Himalayan populations. However, it is not possible to unambiguously rule out one or the other option, since the observed differences between the Himalayan Tibeto-Burman speakers and the Himalayan Indo-European speakers are relatively small.

In addition, some observations cannot easily be explained simply by linguistic or geographical subdivision:

  - When running STRCTURE for K>2 (figure 3B, for K = 4), the Lhokpu, Black Mountain Mönpa and Toto are separated into clusters that are clearly distinct from all other populations. Other than the separation of these three populations, running STRUCTURE for higher numbers of K did not reveal any additional (sub)structure as compared to the observations for K = 2. This absence of additional (sub)structuring for K>2 was also observed in FLOCK 3.0 (figure S2B), although the separation of the Lhokpu, Black Mountain Mönpa and Toto into distinct clusters was much less pronounced in FLOCK.
  - In the MDS plot (figure 4), six Tibeto-Burman populations (Lhokpu, Black Mountain Mönpa, Layap, Kham, Barām and Bhutia) and two Indo-European populations (Tharu and the population-pool of Danuwar & Kachadiya Danuwar) do not cluster together with the majority of the populations belonging to their linguistic phylum.
  - Furthermore, in the MDS plot, the Himalayan populations are not as tightly clustered together as the reference populations.
  - In CHROMA (figure S4), the colour coding patterns for the Tharu and the population-pool of Danuwar & Kachadiya Danuwar are more similar to the pattern observed for most Tibeto-Burman and Altaic populations than to the pattern observed for most Indo-European and Dravidian populations.

Since, due to time-honoured traditions, many of the populations in and around the Greater Himalayan Region tend to marry within their own population or social class, we expected the within population gene identity values to be generally higher than the among population gene identity values. However, as can be seen in figure 4, only a few populations (Orochen-1, Orochen-2, Trung, Lisu, Nù, Barām, Thangmi, Lakha, Layap, Lhokpu, Black Mountain Mönpa and Toto) have relatively high within population gene identity values (>0.25).

The results seen for Tharu and Danuwar & Kachadiya Danuwar may be an indication that these populations, as suspected, do not represent original Indo-European speakers, but rather a group of populations with Tibeto-Burman roots in which the original Tibeto-Burman language has been completely replaced by an Indo-European language.

A possible explanation for the seemingly aberrant behaviour of the other populations mentioned above is that these populations may have undergone recent bottlenecks leading to reduced genetic variation. The assumption that bottlenecks may indeed have occurred seems to be confirmed by the reduced heterozygosity values, reduced average numbers of alleles and higher average relatedness values observed for most of these outlying populations (tables S3A and S3B).

A likely cause for such a bottleneck, at least for Lhokpu, Black Mountain Mönpa and Toto is that these populations are known to have been almost completely isolated from their neighbouring populations until relatively recently, due to both geographical and cultural boundaries. Geographical isolation and subsequent genetic drift could also explain the results observed for the Kham, who live in a very rugged and difficult to negotiate part of Nepal and for the Layap, who live mostly in the relatively inaccessible northernmost part of Bhutan. And for some of the other populations, such as Barām and Thangmi, a certain degree of isolation due to their low social status may have played a part.

However, analysis of the data in Bottleneck 1.2.02 did not indicate a significant recent bottleneck in these populations (data not shown). Furthermore, geographical and/or social isolation cannot be the only explanation for the seemingly aberrant observations, since a certain degree of geographical isolation can be assumed for all Himalayan populations, simply because they live in an area which, historically and even with modern means, is relatively inaccessible. This relative geographical isolation may have resulted in smaller effective population sizes for the Himalayan populations as compared to the populations from surrounding countries, which may explain the less dense clustering pattern the Himalayan populations display in the MDS plot. Alternatively, the observed clustering pattern for the Himalayan populations may lend support to the theory that the Himalayan region has been populated during the early ages of human settlement in Asia, giving the Himalayan populations more opportunity to drift than the populations in the surrounding countries.

Therefore, at present, we do not have a satisfactory explanation for the seemingly aberrant observations, although of course, we cannot rule out the possibility that either the test-populations investigated in this study may not have been adequate representations of the actual populations, e.g. due to too few test-samples or that the set of 15 autosomal STRs chosen in this study does not provide sufficient historical depth to provide a straightforward answer to our problem.

In addition to the analyses discussed above, we performed several Mantel tests in zt, both on the population level and on the individual level. The results of the tests performed across linguistic phyla are summarized in table 3, with r- and p-values of the simple Mantel tests above the diagonal and r- and p-values of the partial Mantel tests below the diagonal. With the results of the previous statistical analyses in mind, it was not unexpected that, at least on the population level, genetic distance shows a significant correlation with both linguistic and geographical distance (these correlations were confirmed by analysis of the data in the IBDWS; results not shown). Also, the significant correlation between geographical distance and linguistic distance was not unexpected, since there is a clear north-south division between the linguistic families included in this project. In zt (and IBDWS, results not shown) the correlation between genetic distance on the one hand and either linguistic distance or geographical distance on the other hand were very similar. It is therefore, not possible, with the current data, to give a clear indication of geography vs linguistics as the major influence on Himalayan genetics. On the individual level, most correlations were not significant, most likely because the dissimilarities based on 15 autosomal STRs are not sufficiently distinctive on this level. However, the general trend observed at the individual level seems to confirm the results observed on the population level.

**Table 3:** Summary of Mantel tests in zt.

	Genetic distance	Linguistic distance	Geographical distance
Genetic distance	-	r_p_ = 0.144210 (p = 0.000010) r_i_ = 0.049613 (p = 0.000100)	r_p_ = 0.134967 (p = 0.012230) r_i_ = 0.009021 (p = 0.030597)
Linguistic distance	r_p_ = 0.115407 (p = 0.000010) r_i_ = 0.049190 (p = 0.000100) *controlled for geographical distance*	-	r_p_ = 0.247616 (p = 0.000010) r_i_ = 0.302360 (p = 0.000100)
Geographical distance	r_p_ = 0.103531 (p = 0.045580) r_i_ = –0.006282 (p = 0.102290) *controlled for linguistic distance*	r_p_ = 0.232692 (p = 0.000010) r_i_ = 0.302297 (p = 0.000100) *controlled for genetic distance*	-

The results of the simple Mantel tests are shown above the diagonal; the results of the partial Mantel tests are shown below the diagonal. r_p_: correlation coefficient for Mantel test on population level with 100 thousand randomisations, r_i_: correlation coefficient for Mantel test on individual level with 10 thousand randomisations. Corresponding p-values are shown between brackets.

For the comparison of genetic distance with both geographical and linguistic distance, the correlation coefficients obtained within linguistic phyla were, on average, approximately 10 times lower than those obtained for the complete dataset and not or only marginally significant. Correlation coefficients for geographical vs linguistic distance were comparable to those obtained for the complete dataset (not shown).

### Concluding remarks

Previous studies, mainly based on the Y-chromosome and/or mtDNA, have indicated the presence of a genetic boundary in Asia, roughly corresponding with the Himalayan mountain range; more recently, some autosomal surveys of Asian populations have also been performed with similar results [4], [6]–[10]. However, none of these studies have so far included a representative sample of the populations residing within the Greater Himalayan Region. With the populations included in the current study, we had hoped to fill this important gap in the Asian genetic survey and to further pinpoint the location of the genetic boundary. However, the results obtained with 15 autosomal STRs are not as clear-cut as those usually obtained with Y-chromosomal and/or mitochondrial analyses: Even though some test results seem to indicate that the influence of language on the current genetic make-up of the Greater Himalayan Region may have been larger than the influence of geography, this cannot be confirmed unambiguously with the analysis methods used. As stated above, this could, at least partially, be due to inadequate population samples or to the small number of STRs used for the comparisons. Thus, comparing genotypes consisting of a larger number of autosomal STRs might improve the picture as might extending the number of population samples examined. Unfortunately, for many of the samples included in this study (especially the reference samples) further genotyping or further sampling is currently not feasible.

Another explanation for our autosomal results being less straightforward could be the dampening effects that recombination and mutation are known to have on the use of autosomal data for deep-rooting analyses. Therefore it is very well possible that the genetic distance based on these 15 autosomal STRs represents a different, probably more recent, historical timeframe than the linguistic distance and the geographical distance. In order to (at least partially) overcome the effect of high mutation rates, markers with a slower mutation rate than that of autosomal STRs can be used. Additionally, the inclusion of Y-chromosomal and mitochondrial data may compensate for the effects of recombination. For that purpose, we decided to initiate a detailed autosomal SNP survey in addition to screening mtDNA and Y-chromosome polymorphisms. This research is still on-going.

## Supporting Information

Figure S1 **Network of Asian languages.** Fitting all populations into this network was not possible with equal branch lengths between all nodes. Therefore, the variation in branch lengths within this network is merely a spatial necessity and does not indicate smaller or larger linguistic distances. In the construction of the linguistic distance matrix, each branch was counted as 1 step, unless indicated differently. Nepalese populations are shown in red, Bhutanese populations in blue, Indian Tibeto-Burman populations in purple, Southern reference populations in green and Northern reference populations in orange, MRCA: Most Recent Common Ancestor, AP: Andhra Pradesh, BR: Bihar, KA: Karnataka, MH: Maharashtra, OR: Orissa, UP: Uttar Pradesh.
(PDF)

Figure S2 **Results of FLOCK 3.0 analyses.** S2A: Results for K = 2. As with the STRUCTURE analyses a clear difference in the clustering patterns for Himalayan Indo-European speakers vs that for Himalayan Tibeto-Burman speakers is visible. S2B: Results for K = 4. In contrast with STRUCTURE, FLOCK does not cluster the Lhokpu, Black Mountain Mönpa and Toto into separate clusters for higher numbers of K. In accordance with STRUCTURE, no further (sub)structuring is observed for the Himalayan populations with higher numbers of K.
(PDF)

Figure S3 **PCoA analysis results.** The colour coding is identical to the colour coding used in the MDS plot. The population-codes used are explained in table 1 and Table S1.
(PDF)

Figure S4 **Results of Chroma analysis.** This figure represents a colour-coded square (pairwise) gene identity matrix (Nei's minimum genetic distance). In general it can be assumed that population pairs showing higher among populations gene identity values (yellow/light orange) are likely to be more closely related than population pairs showing lower among population gene identity values (blue). The diagonal from the lower left corner to the upper right corner represents the within population gene identity values. Populations showing relatively high (>0.25) within population gene identity values are: Orochen-1 (HOR: 0.268), Orochen-2 (ORO: 0.253), Trung (TRU: 0.303), Lisu (LIS: 0.258), Nù (NUB: 0.253), Barām (BAR: 0.261), Thangmi (THG: 0.275), Lakha (LAK: 0.258), Layap (LAY: 0.252), Lhokpu (LHP: 0.288), Black Mountain Mönpa (MON: 0.253) and Toto (TOT: 0.328). Other abbreviations in this figure: DKD: Danuwar & Kachadiya Danuwar, THR: Tahru, AL: Altaic reference populations, TB1: Tibeto-Burman reference populations, TB2: Tibeto-Burman populations from Nepal, TB3: Tibeto-Burman populations from Bhutan, TB4: Tibeto-Burman populations from India, IE1: Indo-European populations from Nepal, IE2: Indo-European reference populations, DR: Dravidian reference populations.
(PDF)

Table S1 **Overview of all reference populations supplied for this study.**
(XLS)

Table S2 **Overview of Nepalese populations pooled for or excluded from statistical analyses.**
(XLS)

Table S3 **Summary statistics.**
(XLS)
