# Supplementary figures and images for: A Linguistically Informed Autosomal STR Survey of Human Populations Residing in the Greater Himalayan Region
Source: PLoS One. 2014 Mar 10;9(3):e91534. doi: 10.1371/journal.pone.0091534 (PMC3948894; doi:10.1371/journal.pone.0091534)

## Figure S1

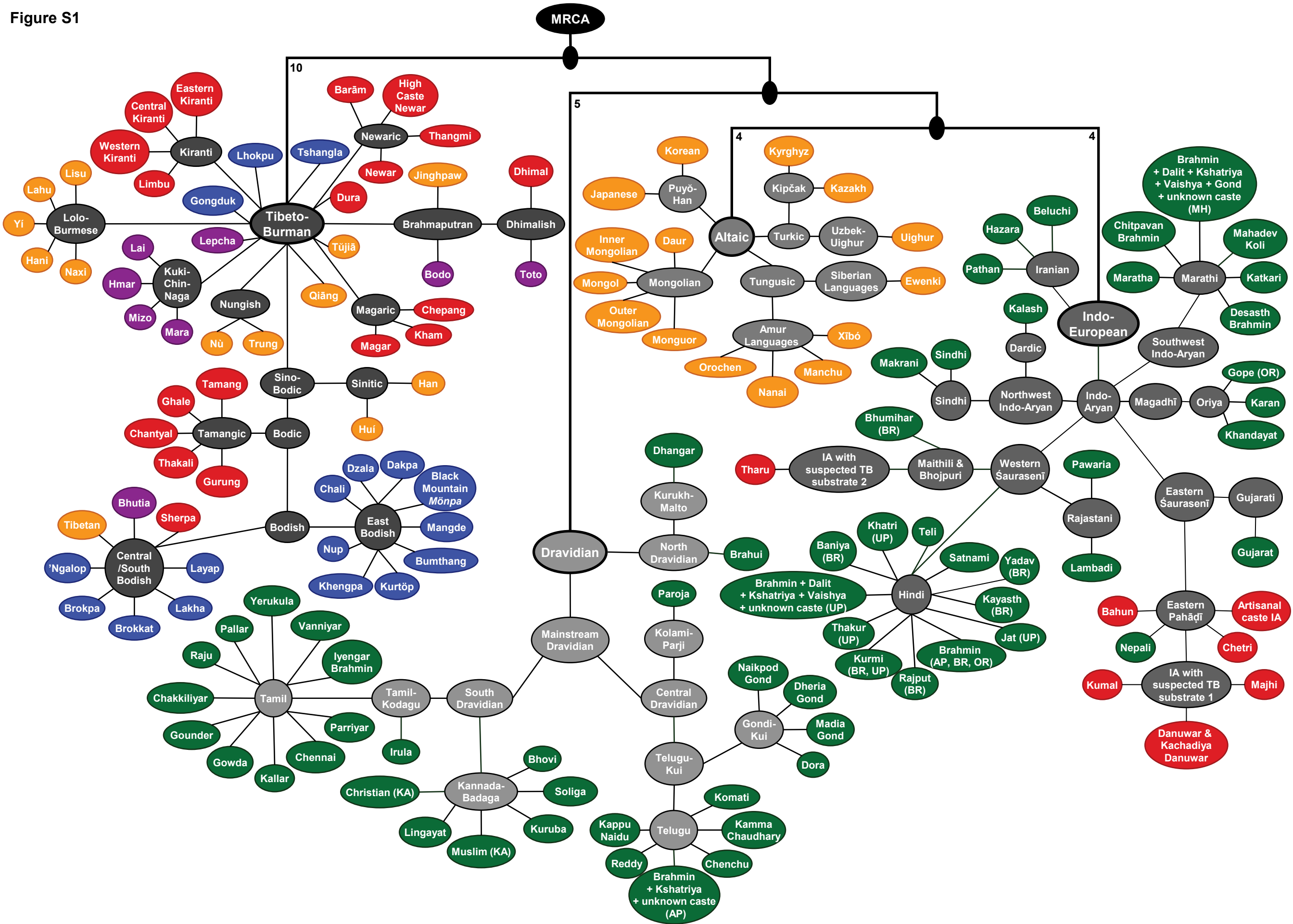

Supplement: Figure S1 — Network of Asian languages. Fitting all populations into this network was not possible with equal branch lengths between all nodes. Therefore, the variation in branch lengths within this network is merely a spatial necessity and does not indicate smaller or larger linguistic distances. In the construction of the linguistic distance matrix, each branch was counted as 1 step, unless indicated differently. Nepalese populations are shown in red, Bhutanese populations in blue, Indian Tibeto-Burman populations in purple, Southern reference populations in green and Northern reference populations in orange, MRCA: Most Recent Common Ancestor, AP: Andhra Pradesh, BR: Bihar, KA: Karnataka, MH: Maharashtra, OR: Orissa, UP: Uttar Pradesh. (PDF) [file pone.0091534.s001.pdf]

Figure S2A

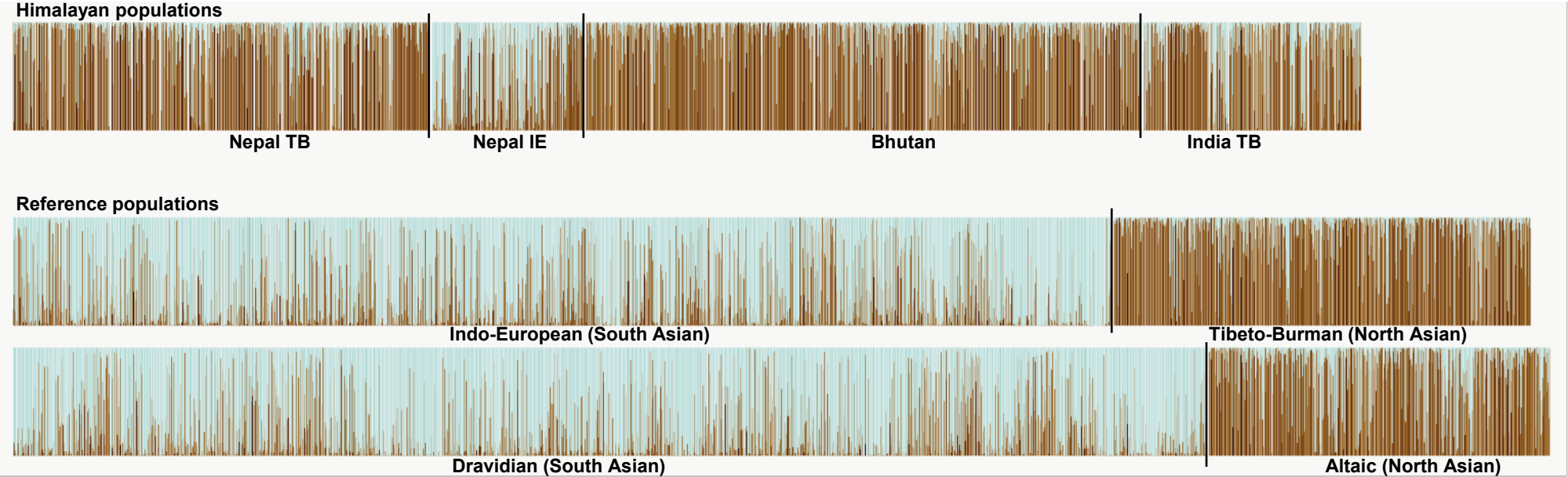

Figure S2B

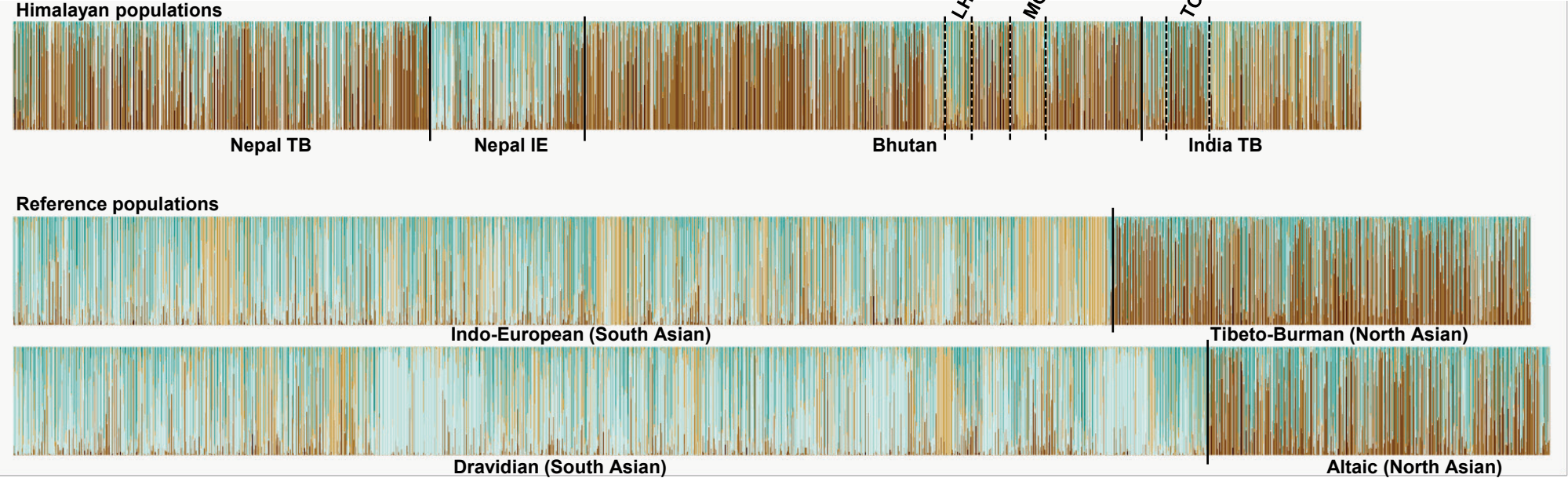

Supplement: Figure S2 — Results of FLOCK 3.0 analyses. S2A: Results for K = 2. As with the STRUCTURE analyses a clear difference in the clustering patterns for Himalayan Indo-European speakers vs that for Himalayan Tibeto-Burman speakers is visible. S2B: Results for K = 4. In contrast with STRUCTURE, FLOCK does not cluster the Lhokpu, Black Mountain Mönpa and Toto into separate clusters for higher numbers of K. In accordance with STRUCTURE, no further (sub)structuring is observed for the Himalayan populations with higher numbers of K. (PDF) [file pone.0091534.s002.pdf]

Figure S3

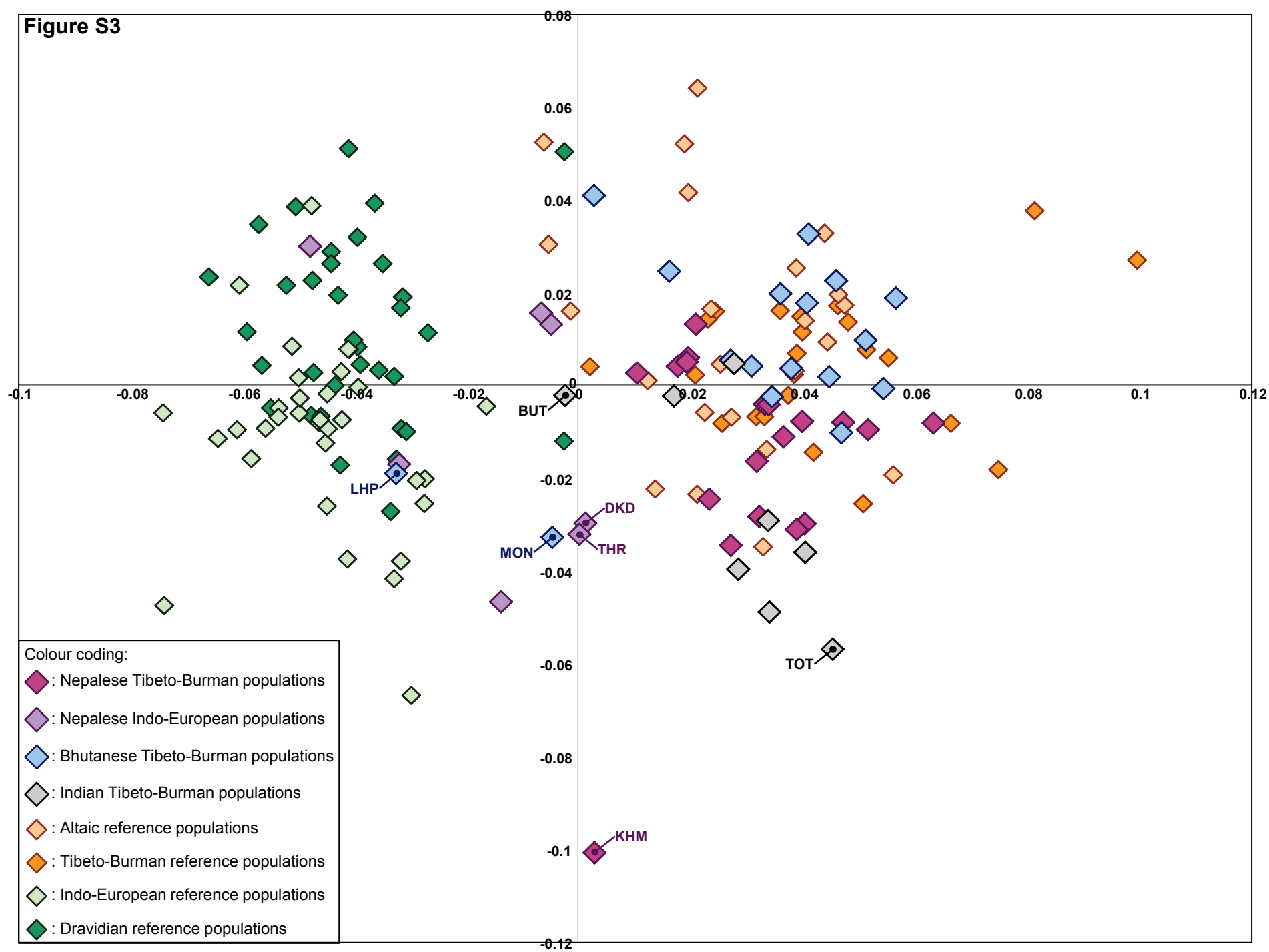

Supplement: Figure S3 — PCoA analysis results. The colour coding is identical to the colour coding used in the MDS plot. The population-codes used are explained in table 1 and Table S1. (PDF) [file pone.0091534.s003.pdf]

Figure S4

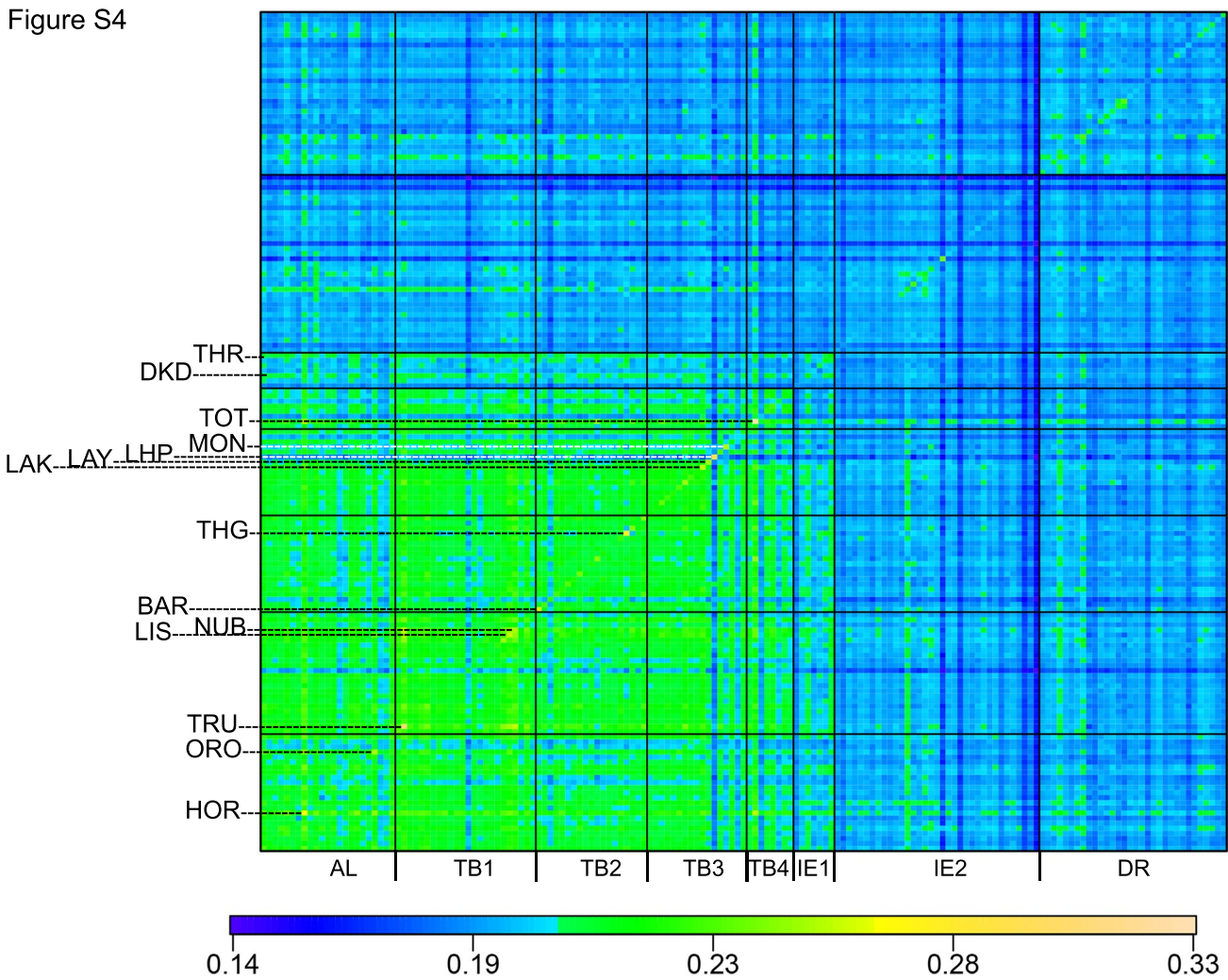

Supplement: Figure S4 — Results of Chroma analysis. This figure represents a colour-coded square (pairwise) gene identity matrix (Nei's minimum genetic distance). In general it can be assumed that population pairs showing higher among populations gene identity values (yellow/light orange) are likely to be more closely related than population pairs showing lower among population gene identity values (blue). The diagonal from the lower left corner to the upper right corner represents the within population gene identity values. Populations showing relatively high (>0.25) within population gene identity values are: Orochen-1 (HOR: 0.268), Orochen-2 (ORO: 0.253), Trung (TRU: 0.303), Lisu (LIS: 0.258), Nù (NUB: 0.253), Barām (BAR: 0.261), Thangmi (THG: 0.275), Lakha (LAK: 0.258), Layap (LAY: 0.252), Lhokpu (LHP: 0.288), Black Mountain Mönpa (MON: 0.253) and Toto (TOT: 0.328). Other abbreviations in this figure: DKD: Danuwar & Kachadiya Danuwar, THR: Tahru, AL: Altaic reference populations, TB1: Tibeto-Burman reference populations, TB2: Tibeto-Burman populations from Nepal, TB3: Tibeto-Burman populations from Bhutan, TB4: Tibeto-Burman populations from India, IE1: Indo-European populations from Nepal, IE2: Indo-European reference populations, DR: Dravidian reference populations. (PDF) [file pone.0091534.s004.pdf]
